# Correlation of *EGFR* or *KRAS* mutation status with ^18^F-FDG uptake on PET-CT scan in lung adenocarcinoma

**DOI:** 10.1371/journal.pone.0175622

**Published:** 2017-04-19

**Authors:** Kazuya Takamochi, Kaoru Mogushi, Hideya Kawaji, Kota Imashimizu, Mariko Fukui, Shiaki Oh, Masayoshi Itoh, Yoshihide Hayashizaki, Weijey Ko, Masao Akeboshi, Kenji Suzuki

**Affiliations:** 1 Department of General Thoracic Surgery, Juntendo University School of Medicine, Tokyo, Japan; 2 Center for Genomic and Regenerative Medicine, Juntendo University School of Medicine, Tokyo, Japan; 3 Preventive Medicine and Applied Genomics Unit, RIKEN Advanced Center for Computing and Communication, Yokohama, Kanagawa, Japan; 4 RIKEN Preventive Medicine and Diagnosis Innovation Program, Wako, Saitama, Japan; 5 Diagnostic Imaging Center, Yotsuya Medical Cube, Tokyo, Japan; University of Nebraska Medical Center, UNITED STATES

## Abstract

**Background:**

^18^F-fluoro-2-deoxy-glucose (^18^F-FDG) positron emission tomography (PET) is a functional imaging modality based on glucose metabolism. The correlation between *EGFR* or *KRAS* mutation status and the standardized uptake value (SUV) of ^18^F-FDG PET scanning has not been fully elucidated.

**Methods:**

Correlations between *EGFR* or *KRAS* mutation status and clinicopathological factors including SUV_max_ were statistically analyzed in 734 surgically resected lung adenocarcinoma patients. Molecular causal relationships between *EGFR* or *KRAS* mutation status and glucose metabolism were then elucidated in 62 lung adenocarcinomas using cap analysis of gene expression (CAGE), a method to determine and quantify the transcription initiation activities of mRNA across the genome.

**Results:**

*EGFR* and *KRAS* mutations were detected in 334 (46%) and 83 (11%) of the 734 lung adenocarcinomas, respectively. The remaining 317 (43%) patients had wild-type tumors for both genes. *EGFR* mutations were more frequent in tumors with lower SUV_max_. In contrast, no relationship was noted between *KRAS* mutation status and SUV_max_. CAGE revealed that 4 genes associated with glucose metabolism (GPI, G6PD, PKM2, and GAPDH) and 5 associated with the cell cycle (ANLN, PTTG1, CIT, KPNA2, and CDC25A) were positively correlated with SUV_max_, although expression levels were lower in *EGFR*-mutated than in wild-type tumors. No similar relationships were noted with *KRAS* mutations.

**Conclusions:**

*EGFR*-mutated adenocarcinomas are biologically indolent with potentially lower levels of glucose metabolism than wild-type tumors. Several genes associated with glucose metabolism and the cell cycle were specifically down-regulated in *EGFR*-mutated adenocarcinomas.

## Introduction

Recently, driver oncogene mutations are being discovered at a rapid pace. Therapeutic agents targeting some of these driver oncogenes have been successfully developed. The somatic mutations in *epidermal growth factor receptor* (*EGFR*) and *v-Ki-ras2 Kirsten rat sarcoma viral oncogene homolog (KRAS)* are the most frequently found in lung adenocarcinomas. The presence of an *EGFR* mutation is the most important predictor of the efficacy of EGFR tyrosine kinase inhibitors (TKIs) [1, 2]. In contrast, *KRAS* mutations are a useful biomarker of EGFR-TKI resistance [3]. It is therefore important to understand the occurrence of *EGFR* and *KRAS* mutations when deciding the initial treatment for lung cancer. However, to obtain sufficient tumor tissue to perform the genetic analyses is frequently difficult in lung cancer patients, especially those with unresectable disease. Non-invasive methods to estimate the probability of the *EGFR*/*KRAS* mutation status are helpful in clinical practice.

^18^F-fluoro-2-deoxy-glucose (^18^F-FDG) positron emission tomography (PET), a functional imaging modality based on glucose metabolism, has become a standard tool for the diagnosis, initial staging, and evaluation of treatment efficacy in lung cancer [4]. High ^18^F-FDG uptake reflects both the increased glucose metabolism and proliferative activity of tumor cells [5, 6]. *EGFR* mutations activate the EGFR-signaling pathway, inhibit apoptosis, and increase cell proliferation, angiogenesis and metastatic potential [7]. *KRAS* plays a key role in the downstream signaling RAS/MAPK pathway of EGFR and other growth factor receptors [7]. Point mutations of *KRAS* also play a critical role in cancer cell growth. Therefore, we hypothesized that there is a causal relationship between increased glucose metabolism and *EGFR* or *KRAS* mutation.

The emergence of next-generation sequencing technologies has enabled a wide range of protocols for more comprehensive and accurate genome-wide analysis. Among these, cap analysis gene expression (CAGE) is a genome-wide approach forming a comprehensive profile of the transcriptome by sequencing only the 5’-ends of capped RNAs [8]. Profiles represent promoter activities based on the frequencies of transcription starting sites (TSSs). CAGE has been used in genome-wide studies such as the ENCODE project [9] and FANTOM5 project [10–12]. Given that the transcriptome represents the molecular basis underlying cellular characteristics, we recently applied CAGE to the study of biomarkers to discriminate distinct types of lung cancer [13].To date, however, CAGE has not been used to study glucose metabolism in tumor cells.

Using transcriptome data from lung adenocarcinomas that monitor expression levels of genes that play important and specific roles in glucose metabolism, we investigated possible correlations between the standardized uptake value (SUV) of ^18^F-FDG PET and *EGFR* or *KRAS* mutation status in lung adenocarcinoma. Furthermore, we also investigated the specific molecular background of glucose metabolism in *EGFR-* or *KRAS*-mutated lung adenocarcinoma.

## Materials and methods

### Patients

Between February 2009 and May 2014, 1414 patients with primary lung cancers, including 1062 with adenocarcinomas, underwent pulmonary resection at our institution. Among these, we retrospectively reviewed 734 adenocarcinoma patients who underwent ^18^F-FDG PET-CT scanning within 2 months before surgery and whose surgically resected specimens were examined for *EGFR* and *KRAS* mutations. Patients who underwent induction chemotherapy and/or radiotherapy were excluded from this study. Patients were classified into three groups according to the mutation status of the tumors, namely *EGFR* mutation-positive (EGFR m^+^), *KRAS* mutation-positive (KRAS m^+^), and wild-type (WT) for both genes. Clinical characteristics such as age, gender, smoking status, preoperative serum carcinoembryonic antigen (CEA) level and SUV_max_ and pathological findings such as tumor size, nodal status, lymphatic permeation and vascular invasion of EGFR m^+^ and KRAS m^+^ tumors were compared to those of WT tumors.

This study was performed using surgical specimens in the tissue bank at our department, which was established with the approval of the institutional review board (IRB) of Juntendo University School of Medicine. Written consent was obtained from all patients prior to surgery for the procurement of tissue for the research purposes. The IRB approved the use of specimens stored in the tissue bank without obtaining new informed consent and deemed that the contents of this study were ethically acceptable.

### ^18^F-FDG PET-CT scanning

As detailed previously [14], PET-CT scan was carried out with a Discovery ST PET/CT scanner (GE Medical Systems; Waukesha, WI, USA) at the Yotsuya Medical Cube (Tokyo Japan). Two experienced nuclear medicine radiologists (W. K. and M. A.) evaluated the PET-CT images, side by side, and reached a consensus on the findings.

### Mutation analyses for *EGFR* and *KRAS*

Genomic DNA was extracted from frozen lung cancer tissues sampled from surgically resected specimens. *EGFR* mutations were analyzed using the peptide nucleic acid-locked nucleic acid polymerase chain reaction (PCR) clamp method [15], and *KRAS* mutations using the peptide nucleic acid-mediated PCR clamping method [16].

### Statistical analysis of the correlations between *EGFR* or *KRAS* mutation status and clinicopathological factors

The Steel-Dwass test was used to compare SUV_max_ among multiple groups based on *EGFR* and *KRAS* mutation patterns. Receiver operating characteristic (ROC) curves were generated to obtain a cut-off for SUV_max_ of the primary tumor which maximizes the sum of sensitivity and specificity for predicting *EGFR* or *KRAS* mutation status. Correlations between *EGFR* or *KRAS* mutation status and clinicopathological factors were evaluated. Univariate analyses between SUV_max_ and each clinicopathological factor were performed by a logistic regression model. All of the variables identified to be significant in the univariate analyses were subsequently entered into the multivariate analyses using a bidirectional (i.e., forward and backward) step-wise logistic regression model. A *P*-value of < 0.05 was considered statistically significant. All statistical analyses were performed using the R statistical software package (version 3.0.2, http://www.r-project.org/).

### CAGE data

CAGE data generated using the previously described protocol [17] were obtained from a previous study [13]. In brief, double-stranded RNA/cDNA produced by reverse transcription from total RNA extracts was purified, oxidized with sodium periodate, and biotinylated with biotin hydrazide. The single-stranded cDNA was recovered after digestion of the single-stranded RNA with RNase I, and ligated with 3’-end and 5’-end adaptors specific to the samples. Double-stranded cDNAs were synthesized and mixed for sequencing in one lane of an Illumina HiSeq2500 sequencer (Illumina; San Diego, CA, USA). The CAGE reads were aligned to the reference genome (hg19) with high mapping quality of ≥ 20.

### Differential and correlation analysis using the CAGE data

The aligned CAGE reads were counted in each region of the FANTOM5 robust peaks [11], a reference set of TSS regions, as raw signals for the promoter activities. Expression (activity) levels of individual promoters were quantified as counts per million (CPM) after normalization by the relative log expression method [18], and subjected to differential analysis using edgeR (version 3.2.4) [19] in R/Bioconductor [20]. Associations between expression levels and SUV_max_ and their statistical significance were assessed by Spearman’s rank correlation. Only results with a false discovery rate (FDR) less than 1% were considered statistically significant, in both the differential and correlation analyses.

## Results

### Patient characteristics and *EGFR* and *KRAS* mutation status

Patient characteristics are summarized in Table 1. Of 734 patients, 367 (50%) were male and 367 (50%) were female. Median age at the time of the operation was 68 years (range, 27–89 years). A total of 363 of 734 (49%) patients were smokers (pack-years > 5) and 371 (51%) were non-smokers (pack-years ≤ 5).

**Table 1:** Clinical characteristics of patients.

Characteristic n (%)		
Age (years)		
	≤ 65	309 (42)
	> 65	425 (58)
Sex		
	Male	367 (50)
	Female	367 (50)
Smoking		
	≤ 5 PY	371 (51)
	> 5 PY	363 (49)
Serum CEA level		
	Normal	386 (53)
	Elevated	348 (47)
Tumor size		
	< 30 mm	514 (70)
	≥ 30 mm	220 (30)
Pathological stage		
	IA/IB	410/123
	IIA/IIB	40/36
	IIIA/IIIB	99/8
	IV	18
Pathological nodal status		
	N0	578 (79)
	N1 / N2	156 (21)
Lymphatic permeation		
	Negative	539 (73)
	Positive	195 (27)
Vascular invasion		
	Negative	514 (70)
	Positive	220 (30)
SUV_max_		
	Median (range)	2.7 (0–33.2)
EGFR mutation		
	Negative	400 (54)
	Positive	334 (46)
	exon 21 L858R	194
	exon 19 deletions	120
	minor mutations	20
KRAS mutation		
	Negative	651 (89)
	Positive	83 (11)
	G to T/G to C	60
	G to A	23

PY = pack years.

Of the 734 lung adenocarcinomas, *EGFR* and *KRAS* mutations were detected in 334 (46%) and 83 (11%), respectively. The EGFR mutation spectra were distributed as follows. The point mutation L858R in exon 21 and deletions in exon 19 were detected in 194 and 120 tumors, respectively, which together accounted for 94% of all *EGFR* alterations. The remaining 6% of the minor *EGFR* mutations were exon 18 G719A in 8 tumors, exon 18 G719S in 5, exon 18 G719C in 2 and exon 21 L861Q in 3. Double mutations were found in 2 tumors; 1 harbored exon 21 L861Q and exon 20 T790M and the other had exon 18 G719A and exon 20 T790M, simultaneously. With regard to *KRAS*, a point mutation in codon 12 was found in 81 (98%) tumors, and a point mutation in codon 13 in 2 (2%). G to T, or G to C transversions were found in 60 (72%) tumors, and G to A transition in 23 (28%). *EGFR* and *KRAS* mutations were mutually exclusive.

**Fig 1:**
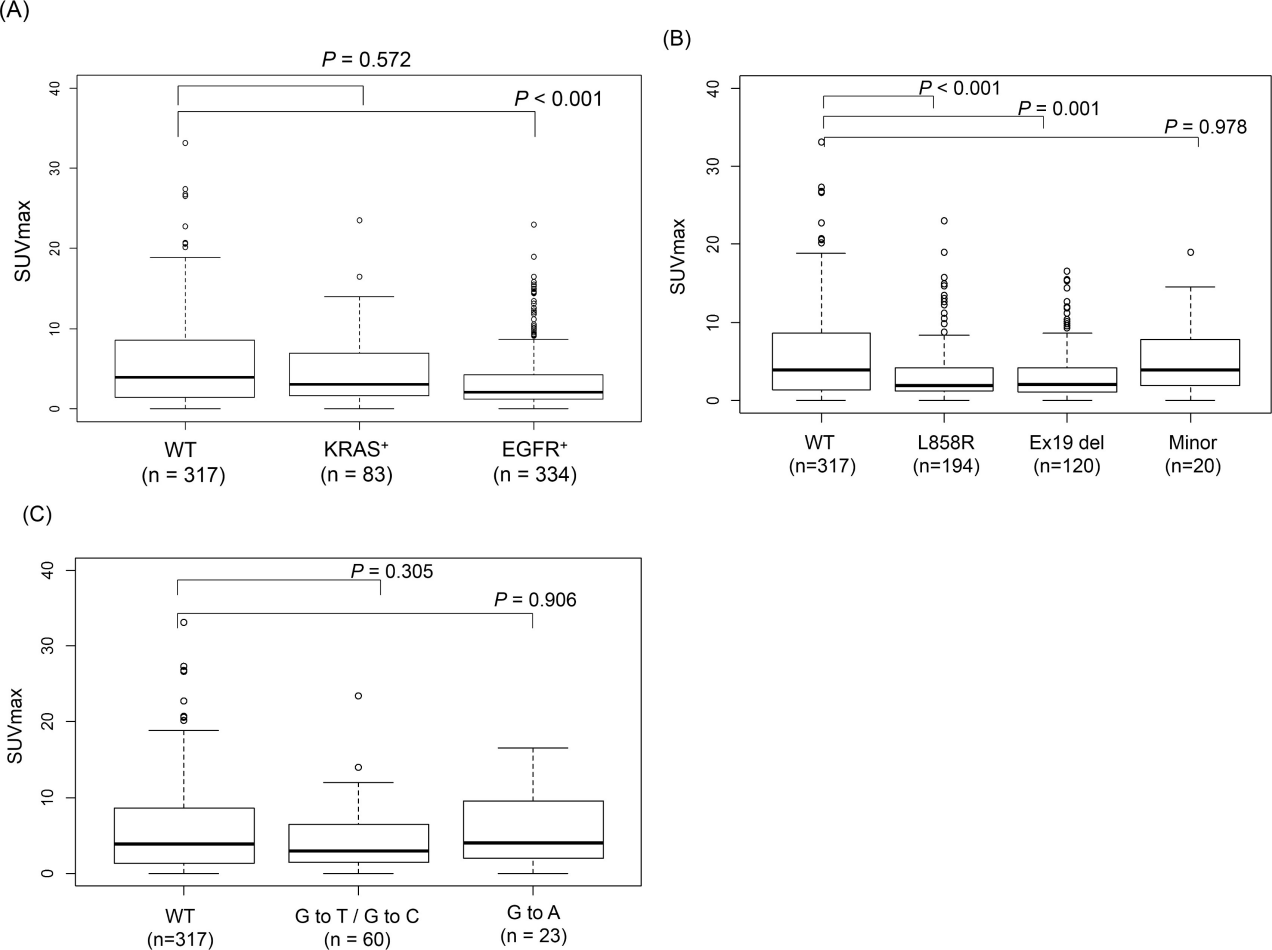
Correlations between SUV_max_ of primary tumors and *EGFR* and *KRAS* mutation status. (A) Box plot of SUV_max_ of primary tumors according to *EGFR* and *KRAS* mutation status, (B) Box plot of SUV_max_ of primary tumors according to *EGFR* mutation spectra, (C) Box plot of SUV_max_ of primary tumors according to *KRAS* mutation spectra.

The median SUV_max_ of all primary tumors was 2.7 (range, 0–33.2). Median SUV_max_ in the EGFR m^+^ group, KRAS m^+^ group, and WT group were 2.1 (range, 0–23), 3.0 (range, 0–23.5), and 3.9 (range, 0–33.2), respectively. SUV_max_ of EGFR m^+^ tumors was significantly lower than that of WT and KRAS m^+^ tumors (Fig 1A). SUV_max_ of tumors with exon 21 L858R or exon 19 deletions was significantly lower than that of WT tumors. However, no significant differences were noted in SUV_max_ between tumors with minor mutations and WT tumors (Fig 1B). The SUV_max_ of KRAS m^+^ tumors did not significantly differ from that of WT tumors (Fig 1A). No significant differences were found in SUV_max_ between tumors with any *KRAS* mutation spectrum (G to T/G to C transversions or G to A transition) and WT tumors (Fig 1C).

### ROC curve analyses of the cut-off values of SUV_max_ for the prediction of *EGFR* or *KRAS* mutations

**Fig 2:**
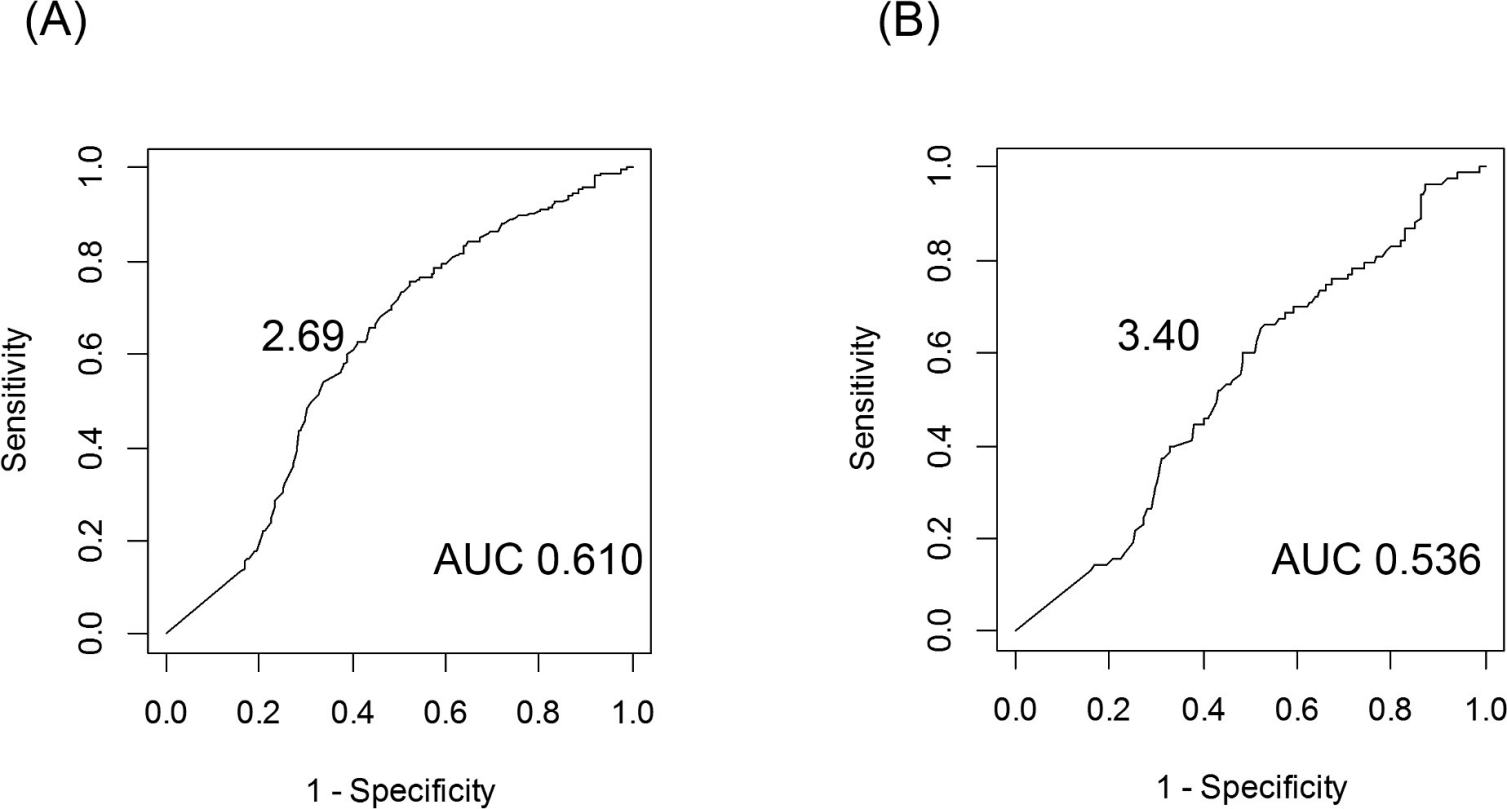
Cut-off values of SUV_max_ in prediction of *EGFR* and *KRAS* mutation. **(A)** *EGFR* mutation, (B) *KRAS* mutation.

Next, we evaluated the prediction of *EGFR* or *KRAS* mutation using SUV_max_. A cut-off value of SUV_max_ ≤ 2.69 provided the highest area under the curve (AUC; 0.610) for predicting *EGFR* mutation, while SUV_max_ ≤ 3.40 provided the highest AUC (0.536) for *KRAS* mutation (Fig 2). Using these cut-off values, parameters for the prediction of *EGFR* mutations were sensitivity, 60%; specificity, 61%; accuracy, 60%; positive predictive value (PPV), 62%; and negative predictive value (NPV), 59%; and parameters for the prediction of *KRAS* mutations were sensitivity, 54%; specificity, 54%; accuracy, 54%; PPV, 23%; and NPV, 82%.

### Univariate and multivariate analysis of the predictors of *EGFR* or *KRAS* mutations

On univariate analysis, *EGFR* mutations were more frequent in females, non-smokers, patients with normal CEA levels, tumors without lymph node involvement or blood vessel invasion, and tumors with lower SUV_max_. On multivariate analysis, significant predictors of *EGFR* mutation were smoking status and SUV_max_ (Table 2). The probability of *EGFR* mutation was inversely correlated with SUV_max._ Univariate analyses showed that *KRAS* mutations were more frequent in males and smokers. On multivariate analysis, the only significant predictor of *KRAS* mutation was smoking history (Table 3). No relationship was found between the *KRAS* mutation status and SUV_max_. The predictability of *EGFR* mutation status was compared between combinations of well-established clinical factors with or without SUV_max_ (Table 4). PPV of *EGFR* mutation status was increased by adding SUV_max_ to gender and smoking status.

**Table 2:** Univariate and multivariate analysis of predictors of *EGFR* mutation.

Characteristic		WT	EGFR m^+^	Univariate analysis		Multivariate analysis	
		(n = 317)		Odds ratio (95% CI)	*p*-value	Odds ratio (95% CI)	*p*-value
Age (years)							
	≤ 65	143	137	1			
	> 65	174	197	1.182 (0.866–1.613)	0.292		
Sex							
	Female	136	210	1			
	Male	181	124	0.444 (0.323–0.607)	< 0.001		
Smoking							
	≤ 5 PY	131	229	1		1	
	> 5 PY	186	105	0.323 (0.234–0.444)	< 0.001	0.357 (0.256–0.494)	< 0.001
Serum CEA level							
	Normal	157	197	1			
	Elevated	160	137	0.682 (0.500–0.930)	0.016		
Tumor size							
	< 30 mm	218	243	1			
	≥ 30 mm	99	91	0.825 (0.587–1.156)	0.264		
Pathological nodal status							
	N0	232	277	1			
	N1 / N2	85	57	0.562 (0.383–0.818)	0.003		
Lymphatic permeation							
	Negative	221	253	1			
	Positive	96	81	0.737 (0.521–1.041)	0.084		
Vascular invasion							
	Negative	204	251	1			
	Positive	113	83	0.597 (0.425–0.836)	0.003		
SUV_max_							
	≤ 2.69	124	200	1		1	
	> 2.69	193	134				

WT = wild-type; m^+^ = mutation-positive; PY = pack years.

**Table 3:** Univariate and multivariate analysis of predictors of *KRAS* mutation.

Characteristic		WT	KRAS m^+^	Univariate analysis		Multivariate analysis	
		(n = 317)	(n = 83)	Odds ratio (95% CI)	*p*-value	Odds ratio (95% CI)	*p*-value
Age (years)							
	≤ 65	143	29	1			
	> 65	174	54	1.530 (0.932–2.554)	0.097		
Sex							
	Female	136	21	1			
	Male	181	62	2.218 (1.308–3.890)	0.004		
Smoking							
	≤ 5 PY	131	12	1		1	
	> 5 PY	186	71	4.167 (2.248–8.359)	< 0.001	4.167 (2.248–8.359)	< 0.001
Serum CEA level							
	Normal	157	32	1			
	Elevated	160	51	1.564 (0.959–2.581)	0.076		
Tumor size							
	< 30 mm	218	53	1			
	≥ 30 mm	99	30	1.246 (0.745–2.059)	0.394		
Pathological nodal status							
	N0	232	69	1			
	N1 / N2	85	14	0.554 (0.286–1.009)	0.064		
Lymphatic permeation							
	Negative	221	65	1			
	Positive	96	18	0.637 (0.351–1.112)	0.124		
Vascular invasion							
	Negative	204	59	1			
	Positive	113	24	0.734 (0.427–1.231)	0.251		
SUV max							
	≤ 3.4	147	45	1			
	> 3.4	170	38	0.730 (0.448–1.185)	0.204		

WT = wild-type; m^+^ = mutation-positive; PY = pack years.

**Table 4:** Predictability of the *EGFR* mutation status by the combinations of well-established clinical factors with or without SUV_max_.

		EGFR mutation status		Sensitivity	Specificity	PPV	NPV	Accuracy
Clinical predictors		Positive	Negative					
Female & Non-smoker *	Yes	182	115	54%	71%	61%	65%	64%
	No	152	285					
Non-smoker & SUV_max_ ≤ 2.69	Yes	131	83	39%	79%	61%	61%	61%
	No	203	317					
Female & Non-smoker	Yes	110	66	33%	84%	63%	60%	60%
& SUV_max_ ≤ 2.69	No	224	334					

* means pack-years ≤ 5.

PPV = positive predictive value; NPV = negative predictive value.

### CAGE for the molecular background of glucose metabolism in *EGFR* or *KRAS* mutated lung adenocarcinoma

**Fig 3:**
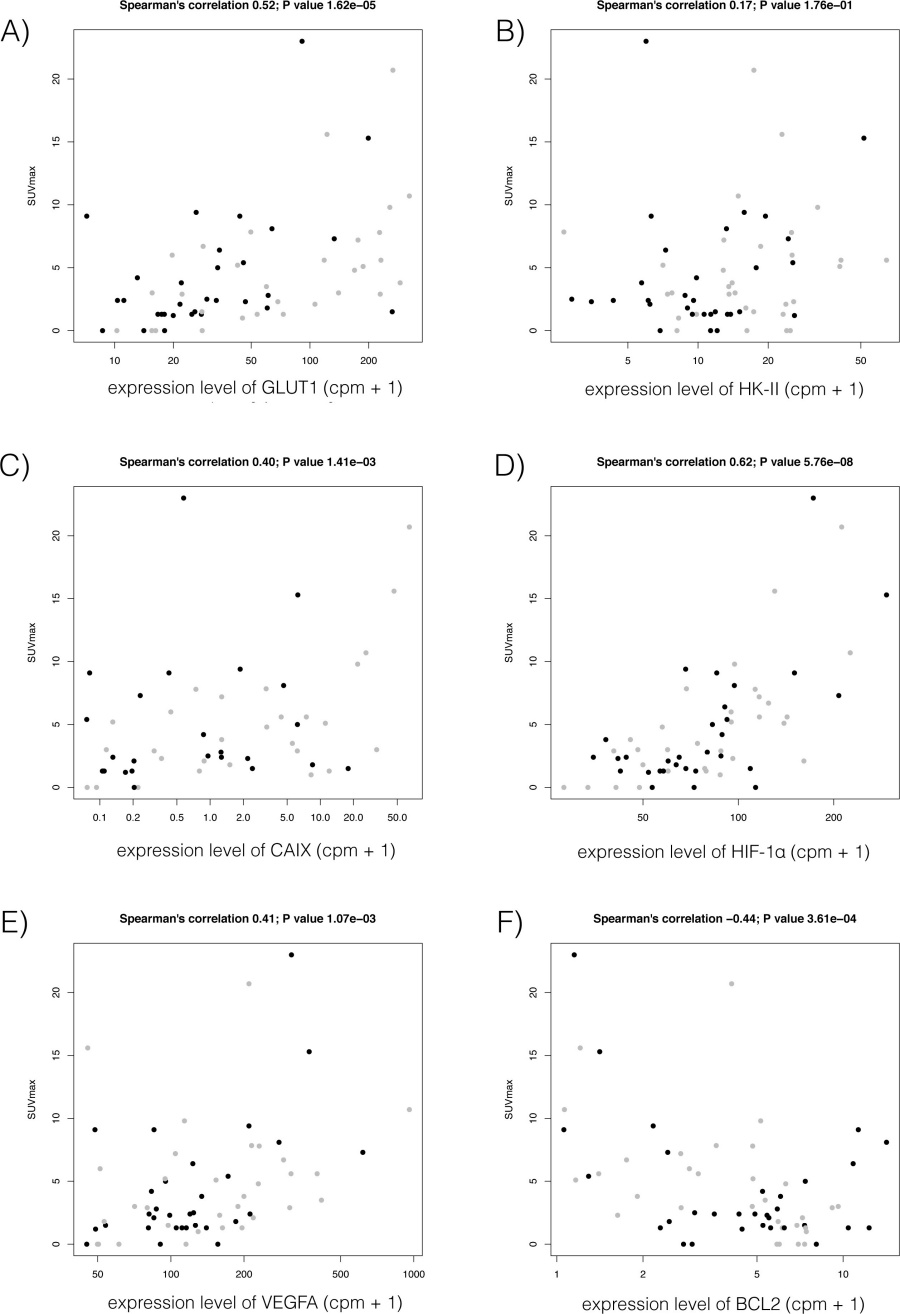
**Scatter plots of association of SUV**_**max**_ **with expression levels of four genes associated with glucose metabolism (A-D) and two genes associated with cell proliferation (E and F): (A) GLUT-1, (B) HK-II, (C) CAIX, (D) HIF-1α, (E) VEGF, and (F) BCL2.** Y-axis represents SUV_max_ and X-axis represents gene expression monitored by CAGE, in which the most correlated promoter activities are shown. Black and gray dots represent donors with *EGFR* mutation-positive (EGFR m^+^) and wild-type, respectively.

Further, we examined expression levels of genes based on the CAGE results (Takamochi et al., submitted), in particular those related to glucose metabolism and the cell cycle, in association with SUV_max._ We manually selected 7 genes associated with glucose metabolism: class I glucose transporters (GLUT1, GLUT2, GLUT3, GLUT4), hexokinase-II (HK-II), hypoxia-inducible factor-1 alpha (HIF-1α), and carbonic anhydrase IX (CAIX). Of these, 4 genes (GLUT1, HK-II, HIF-1α, and CAIX) showed positive correlations between their expression levels monitored by CAGE with SUV_max_ across 62 lung adenocarcinomas (Fig 3). Next, we selected 5 genes associated with cell growth: TP53, CCND1, BCL2, vascular endothelial growth factor (VEGF), and MKI67. Of these, expression of VEGF showed a positive correlation with SUV_max_, while BCL2 showed an inverse correlation with SUV_max_ (Fig 3).

**Fig 4:**
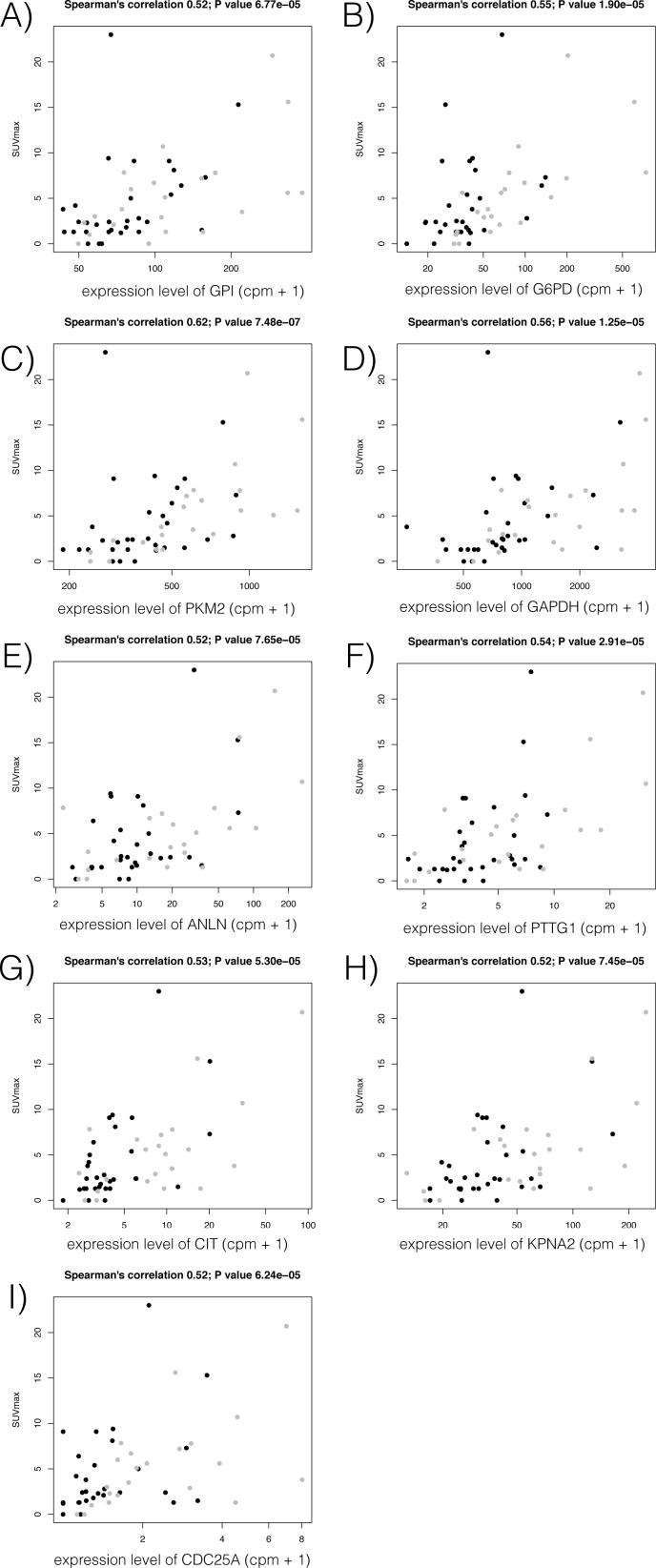
**Association of SUV**_**max**_ **with expression levels of genes associated with glucose metabolism (A-D) or the cell cycle (E-I), which were specifically down-regulated in *EGFR*-mutated tumors compared to wild-type tumors and correlated with SUV**_**max**_**: (A) GPI, (B) G6PD, (C) PKM2, (D) GAPDH, (E) ANLN, (F) PTTG1, (G) CIT, (H) KPNA2, and (I) CDC25A.** Y-axis represents SUV_max_ and X-axis represents gene expression monitored by CAGE, showing the most correlated promoter activities. Black and gray dots represent donors with *EGFR* mutation-positive (EGFR m^+^) and wild-type, respectively.

We expanded this expression analysis to examine genes involved in the 2 pathways. Among genes whose promoters were more significantly down-regulated in EGFR m^+^ tumors than WT tumors (FDR < 1%), we found that both glucose metabolism-related and cell cycle-related genes were enriched (P value < 5.2e-18 and 0.02, with GO term enrichment analysis with DAVID) [21, 22]. Of these, 4 genes associated with glucose metabolism (GPI, G6PD, PKM2, and GAPDH) and 5 genes associated with the cell cycle (ANLN, PTTG1, CIT, KPNA2, and CDC25A) showed a positive correlation between expression and SUV_max_. (FDR < 1%; Fig 4). Notably, none of the genes down-regulated in KRAS m^+^ tumors showed significant correlation with SUV_max_.

## Discussion

In this study, we found that the probability of EGFR mutation in lung adenocarcinoma was inversely correlated with SUV_max_. In contrast, the probability of KRAS mutation was not correlated with SUV_max_. Further, several genes associated with glucose metabolism or the cell cycle were specifically down-regulated in EGFR m^+^ adenocarcinomas. These findings suggest that EGFR m^+^ adenocarcinomas are biologically indolent with potentially lower levels of glucose metabolism than wild-type tumors.

To our knowledge, this is the largest study to evaluate the correlations between ^18^F-FDG uptake and *EGFR* mutation status in lung cancer, and the first to investigate the correlation between the ^18^F-FDG uptake and *KRAS* mutation status. The 4 retrospective studies that previously investigated the correlation between the ^18^F-FDG uptake and *EGFR* mutation status in lung cancer [23–26] reported contradictory findings (Table 4). In their multivariate analysis, Huang et al.[23] and Ko et al.[26] showed that a higher SUV_max_ was a significant predictor of *EGFR* mutation, whereas Na et al.[25] and Mak et al.[24] reported that a lower SUV_max_ of the primary tumor was predictive of *EGFR* mutation. Our findings are compatible with those of the latter groups [24, 25]. These conflicting results may have resulted from differences in the ethnic background or the small size of the study populations (Table 5).

**Table 5:** Clinical studies of the role of ^18^F-FDG uptake on PET-CT scans in predicting *EGFR* mutation status.

Author/year	Ethnicity	No. of patients	Histology	Stage	EGFR mutation	Results *
Huang et al./2010	Asian (Taiwanese)	77	Ad	Clinical IIIB or IV	49 (64%)	SUV_max_ ≥ 9.5, EGFR m^+^ 78%
Na et al./2010	Asian (Korean)	100	53 Ad, 47 non-Ad	Pathological I-IV	21 (21%)	SUV_max_ < 9.2, EGFR m^+^ 40%
Mak et al./2011	White (88% of all)	100	90 Ad, 10 non-Ad	Clinical I-IV	24 (24%)	SUV_max_ ≥ 5.0, WT 96%
Ko et al./2014	Asian (Taiwanese)	132	Ad	Clinical I-IV	69 (52%)	SUV_max_ ≥ 6.0, EGFR m^+^63%
Present study	Asian (Japanese)	734	Ad	Pathological I-IV	334 (46%)	SUV_max_ ≤ 2.69, EGFR m^+^ 62%

* shows threshold SUV_max_ and positive predictive value of EGFR mutation status.

Ad = adenocarcinoma; m^+^ = mutation-positive; WT = wild-type.

Consistent with numerous previous reports [27–29], *EGFR* mutations in the present study were more frequent in females and never-smokers. In addition, a higher probability of *EGFR* mutation was observed in tumors without lymph node involvement or blood vessel invasion and in those with a lower SUV_max_. Higashi et al.[30] reported that the prevalence rates of lymphatic permeation and lymph node involvement were lower in primary tumors with low ^18^F-FDG uptake than those with a higher ^18^F-FDG uptake. These findings suggest that EGFR m^+^ adenocarcinomas are biologically indolent with potentially lower levels of glucose metabolism.

Although many factors have been reported to influence ^18^F-FDG uptake, the precise biological mechanism by which ^18^F-FDG accumulates in malignant cells remains to be clarified. In 1985, Mueckler et al.[31] initially reported that facilitative glucose transport across the plasma membrane was mediated by a family of structurally related proteins known as facilitated diffuse GLUTs. Among the 14 currently known GLUT isoforms [32], the overexpression of GLUT-1 has been shown to be most closely related to ^18^F-FDG uptake in lung cancer [33–35]. Sasaki et al.[36] reported that GLUT-1 overexpression evaluated by immunohistochemistry was significantly correlated with *EFGR* or *KRAS* mutation status, with overexpression in 18 (24%) of 76 EGFR m^+^ lung cancers and 20 (67%) of 30 KRAS m^+^ lung cancers. In our present patients, we found that the expression level of GLUT-1 was positively correlated with SUV_max_, as were other genes related to glucose metabolism, namely HK-II, CAIX, and HIF-1α (Fig 3). This finding is consistent with previous reports [34, 37]. GO term analysis revealed that the glucose metabolism-related and the cell cycle-related genes were enriched among the down-regulated genes in EGFR m^+^ adenocarcinomas, which supports our results for ^18^F-FDG PET, with lower levels of SUV_max_. Notably, 4 of the glucose metabolism-related genes, GPI, G6PD, PKM2, and GAPDH and 5 of the cell cycle-related genes, ANLN, PTTG1, CIT, KPNA2, and CDC25A, were significantly down-regulated in EGFR m^+^ adenocarcinomas, and showed a substantial correlation with SUV_max_ (Fig 4). These likely comprise a common subset of the pathway underlying EGFR mutation and glucose metabolism.

Several limitations of our study warrant mention. First, it was conducted under a retrospective design in patients who required surgical resection, most for early stage disease. Accordingly, the selected cases might not have reflected the overall features of lung adenocarcinoma. Second, the sample size of KRAS m^+^ tumors was too small to allow any firm conclusions. Although we found no significant relationship between ^18^F-FDG uptake and *KRAS* mutation status in lung adenocarcinoma and did not identify any genes specifically correlated with glucose metabolism in KRAS m^+^ tumors, a conclusive answer to this question would require a larger sample size.

In summary, the probability of *EGFR* mutation was inversely correlated with SUV_max_. In contrast, the probability of *KRAS* mutation was not correlated with SUV_max_. Several genes associated with glucose metabolism or the cell cycle were specifically down-regulated in EGFR m^+^ adenocarcinomas. These findings confirm that EGFR m^+^ adenocarcinomas are biologically indolent with potentially lower levels of glucose metabolism than wild-type tumors.
